# Effectiveness of guselkumab for avelumab‐induced psoriasis in urothelial carcinoma: A case report

**DOI:** 10.1111/1346-8138.17583

**Published:** 2024-12-12

**Authors:** Kazuki Yatsuzuka, Satoshi Yoshida, Noriyoshi Miura, Nobushige Kohri, Jun Muto, Ken Shiraishi, Yasuhiro Fujisawa

**Affiliations:** ^1^ Department of Dermatology Ehime University Graduate School of Medicine Toon Ehime Japan; ^2^ Department of Urology Ehime University Graduate School of Medicine Toon Ehime Japan

Dear Editor,

Immune checkpoint inhibitors (ICIs) have revolutionized cancer treatment; however, their use is often accompanied by immune‐related adverse events, including skin manifestations. Although topical corticosteroids are typically effective, systemic therapies are sometimes required. In severe cases, ICIs may need to be withheld.^1^ ICI‐mediated psoriasis (ICMP), characterized by new‐onset or worsening psoriasis, is a recognized adverse effect of ICI.^2^ Recent studies have demonstrated the successful management of ICMP using biologics without ICI discontinuation.^3^ We present a case of ICMP effectively treated with biologics while continuing ICI therapy.

A 59‐year‐old man presented with widespread erythematous plaques. Despite achieving near‐complete remission of psoriasis vulgaris with topical steroids for the past 6 months, he developed erythematous plaques with infiltration and scaling on his face, extremities, and trunk within a month of initiating avelumab for advanced urothelial carcinoma (Figure 1a,b). A skin biopsy revealed hyperkeratosis, parakeratosis, elongation of the rete ridges, and neutrophil infiltration into the epidermis (Figure 1c,d). We diagnosed a flare‐up of psoriasis vulgaris induced by avelumab and temporarily suspended avelumab despite its oncological efficacy. Psoriasis area and severity index (PASI) score at flare‐up was 13.2. Since the eruptions were resistant to very strong topical steroids, we added narrowband UV‐B therapy and the PASI score improved to 2.1 after 3 weeks. Although avelumab was reintroduced, the PASI score worsened to 8.4 within a month. After the addition of apremilast, the PASI score decreased to 1.4. However, 10 months after apremilast initiation, a third flare‐up occurred, with the PASI score escalating to 9.6 (Figure 1e,f). Considering the sustained partial response of urothelial carcinoma to avelumab, we decided to switch psoriasis treatment to guselkumab while continuing avelumab. Consequently, a 50% PASI improvement was observed at 12 weeks (Figure 1g,h), with further improvement to a PASI score of 1.2 at 28 weeks without AEs. His urothelial cancer remains under control with continued avelumab.

**FIGURE 1:**
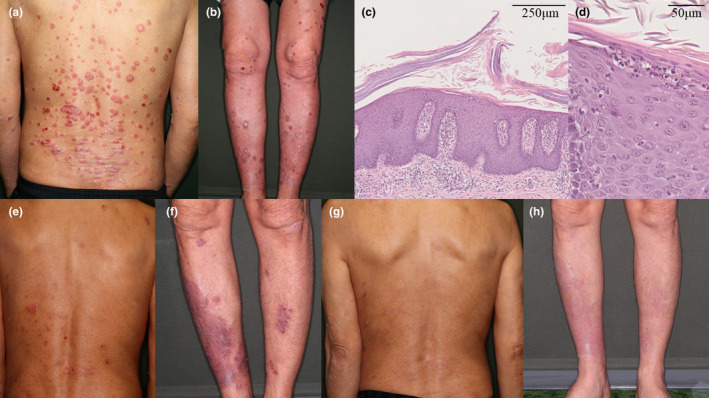
(a, b) Erythematous plaques with infiltration and scaling developed on the patient's face, extremities, and trunk 1 month after initiation of avelumab. (c, d) The histopathological findings of a plaque show hyperkeratosis, parakeratosis, granular layer thinning, rete ridge elongation, and neutrophil infiltration into the epidermis. (e, f) Despite the addition of apremilast and UV therapy to avelumab, the eruptions worsened 10 months later. (g, h) Following guselkumab initiation, a 50% reduction in the psoriasis area and severity index score was achieved at 12 weeks.

Topical agents are the mainstay of treatment for ICMP. Switching to a different class of ICI should also be considered. Nikolaou et al.^2^ proposed algorithm‐based management strategies. Although their algorithm prioritizes ICI continuation, a significant proportion of their cohort (18%) required permanent ICI discontinuation because of psoriasis.^2^ Recent studies have emphasized the efficacy and safety of biologics, particularly interleukin (IL) 23 and IL‐17 inhibitors, in the management of psoriasis concurrently with cancer treatment.^4^ Studies have shown that reduced tumor expression of psoriasis pathway mediators such as IL‐17A and IL‐23A do not affect overall survival for most tumor types.^2^ Recent case reports have demonstrated successful psoriasis management with biologics in patients receiving ICIs without compromising antitumor efficacy.^3^, ^5^ To our knowledge, this is the first successful use of guselkumab for avelumab‐induced psoriasis in a patient with urothelial carcinoma without discontinuing ICI. Considering the pivotal role of ICIs in oncology, the judicious use of biologics should be considered as a primary therapeutic strategy for psoriasis in this patient population rather than premature ICI discontinuation. However, the combination of ICIs and biologics might complicate the immune system, increase the risk of immune‐related adverse events and opportunistic infections, and require careful follow‐up.

## CONFLICT OF INTEREST STATEMENT

Kazuki Yatsuzuka has received speaker's fees from Abbvie, Eli Lilly, Janssen, Maruho, Novartis, Sun pharma, Taiho, and UCB; and has received research grants from Sun pharma outside the submitted work. Jun Muto has received speaker's fees from Abbvie, Eli Lilly, and Maruho; and has received research grants from Rohto outside the submitted work. Satoshi Yoshida, Noriyoshi Miura, Nobushige Kohri, Ken Shiraishi, and Yasuhiro Fujisawa have declared no conflict of interest. Yasuhiro Fujisawa is an editorial board member of the *Journal of Dermatology* and a co‐author of this article. To minimize bias, he was excluded from all editorial decision‐making related to the acceptance of this article for publication.

## INFORMED CONSENT

We obtained written informed consent from the patient to publish his clinical details.
